# A New MicroRNA Cluster Involved in the Reprogramming to a Pluripotent State

**DOI:** 10.32607/20758251-2019-11-2-92-97

**Published:** 2019

**Authors:** V. V. Sherstyuk, G. I. Davletshina, Y. V. Vyatkin, D. N. Shtokalo, V. V. Vlasov, S. M. Zakian

**Affiliations:** The Federal Research Center Institute of Cytology and Genetics SB RAS, Lavrentyeva Ave. 10, Novosibirsk, 630090, Russia; E.Meshalkin National medical research center Ministry of Healthcare of the Russian Federation, Rechkunovskaya Str. 15, Novosibirsk, 630055, Russia; Novosibirsk State University, Pirogova Str. 2, Novosibirsk, 630090, Russia; Institute of Chemical Biology and Fundamental Medicine SB RAS, Lavrentyeva Ave. 8, Novosibirsk, 630090, Russia; AcademGene LLC, Lavrentyeva Ave. 6, Novosibirsk, 630090, Russia; St. Laurent Institute, New Boston St., 317, 01801, Woburn, MA, USA; A.P.Ershov Institute of Informatics Systems SB RAS, Lavrentyeva Ave. 6, Novosibirsk, 630090, Russia

**Keywords:** microRNA, pluripotent stem cells, reprogramming, CRISPR/Cas9

## Abstract

Reprogramming of somatic cells to a pluripotent state is a complex, multistage process that is regulated by many factors. Among these factors, non-coding RNAs and microRNAs (miRNAs) have been intensively studied in recent years. MiRNAs play an important role in many processes, particularly in cell reprogramming. In this study, we investigated the reprogramming of rat fibroblasts with a deleted locus encoding a cluster comprising 14 miRNAs (from miR-743a to miR-465). The deletion of this locus was demonstrated to decrease significantly the efficiency of the cell reprogramming. In addition, the cells produced by the reprogramming differed from rat embryonic and induced pluripotent stem cells, which was an indication that reprogramming in these cells had not been completed. We suggest that this miRNA cluster or some of its members are involved in regulating the reprogramming of rat cells to a pluripotent state.

## INTRODUCTION

Pluripotent stem cells are cells capable of differentiating into derivatives of all three germ layers. One of the ways to produce pluripotent stem cells is to reprogram somatic cells by overexpressing Oct4, Sox2, Klf4, and c-Myc pluripotency factors [1]. This process results in the so-called induced pluripotent cells (iPSCs) that are widely used for studying early developmental and differentiation processes and modeling hereditary diseases and are a promising source of the cellular derivatives used in regenerative medicine. The reprogramming mechanisms have been well studied, and the changes in gene expression, chromatin organization, and metabolism are known. In addition, this process involves microRNAs (miRNAs) that are a class of small non-coding RNAs, from 18 to 23 nucleotides in length, that participate in post-transcriptional regulation of gene expression. MiRNAs play an important role in the regulation of various processes, including organism development and cell differentiation. To date, many miRNAs expressed in human, mouse, and rat pluripotent stem cells are known. The most studied miRNAs involved in the reprogramming process belong to the miR-290-295 and miR-302-367 clusters and miR-200 family [2]. However, many other miRNAs are involved in cell reprogramming as well; their functions remain unknown. Earlier, we analyzed the expression of miRNAs in rat embryonic stem cells (ESCs), iPSCs, and embryonic fibroblasts and identified a miRNA cluster on the X chromosome (from miR-743a to miR-465) which was characterized by an increased expression level in pluripotent cells compared to that in fibroblasts [3]. In addition, expression of some miRNAs in this cluster decreases during spontaneous differentiation of pluripotent cells. Our findings suggest that these miRNAs may be involved in the processes of self-renewal and pluripotent state maintenance in stem cells, as well as in their reprogramming. To investigate the involvement of these miRNAs in the reprogramming process, we obtained rat fibroblasts carrying a deletion of the genome region encoding the miRNAs under study. Deletion of this region disrupts reprogramming to a pluripotent state, which indicates involvement of this miRNA cluster or some of its members in the regulation of the reprogramming process.

## EXPERIMENTAL

Guide RNAs flanking a target miRNA cluster were selected using the Benchling online platform (https://benchling.com/crispr). We chose the following protospacers: 5’-CTTAGTTAACAGATTAGGAC-3’ (PAM-TGG) and 5’-TTGCTAGAGTAATACCAACT-3’ (PAM-TGG). The oligonucleotides were inserted into the pX-458-2sgRNA vector at the BbsI and BsaI sites. The pX-458-2sgRNA vector was obtained by hydrolysis of the pX333 vector (Addgene Plasmid #64073) by XbaI and KpnI restriction endonucleases, isolation and purification of a 444 bp fragment, and insertion of the fragment into the pSpCas9(BB)-2A-GFP (PX458) vector (Addgene Plasmid #48138) hydrolyzed by XbaI and KpnI.

Rat fibroblasts were cultured at 37 °C and 5% CO_2_ in a 1:1 mixture of DMEM and F12 (Lonza) media supplemented with 10% fetal bovine serum (Gibco, USA), GlutaMAX (Gibco), and a mixture of 100 U/mL penicillin and 100 μg/mL streptomycin (Gibco). To obtain the deletion, fibroblasts (4 × 105) of male rats were electroporated with the pX-458-2sgRNA plasmid (5 μg) containing cloned RNA guides using a Neon Transfection System device (Invitrogen, USA). On the next day, the cells were sorted using a S3e Cell Sorter (Bio-Rad, USA) and subcloned into 96-well plates. After 7–14 days, the wells were examined under a microscope and those containing several growth islands were discarded to exclude polyclonal lines. Monoclonal lines were propagated, and the DNA was isolated and analyzed by PCR and Sanger sequencing. Primer sequences are given in *Table 1*.

**Table 1:** Primer sequences for the PCR analysis of cell lines with a deletion of the target locus

Primer	Sequence, 5’–3’
FL1	CATACCTCAGAAACGCAAAAC
FL2	AGTTAATATCGAAAAGCCACC
IN1	CAGAATATATGGCTTATTGGA
IN2	GTTTTATACATACGCACACC
IN3	TATAAGAATGAAAGACGCCAAAC

For reprogramming, fibroblasts (5 × 104) were transduced with two samples of lentiviruses encoding Oct4, Sox2, Klf4, and c-Myc pluripotency factors and the tetracycline transactivator. One hour before transduction, 4 μg/mL polybrene (Sigma-Aldrich, USA) was added to the medium. Lentivirus samples were prepared using TetO-FUW-OSKM (Addgene Plasmid #20321) and FUdeltaGW-rtTA (Addgene Plasmid #19780) vectors and vectors encoding viral packaging proteins, psPAX2 (Addgene Plasmid #12260) and pMD2.G (Addgene Plasmid #12259), according to a protocol described elsewhere [4]. The next day after transduction, 2 μg/mL doxycycline (Sigma-Aldrich) was added to the medium; on the fourth day, the fibroblasts were plated onto a layer of mitotically inactive mouse embryonic fibroblasts and cultured in a N2B27 medium consisting of a N2 (DMEM/F12 with addition of N2) (Gibco) and B27 (Neurobasal with addition of B27) (Gibco) mixture, GlutaMAX, a mixture of 100 U/mL penicillin and 100 μg/mL streptomycin, 0.1 mM 2-mercaptoethanol (Sigma-Aldrich), 1,000 U/mL mouse LIF (StemRD), 1 μM PD0325901 (StemRD), and 3 μM CHIR99021 (StemRD). Reprogramming was performed in triplicate. On days 10–14 of reprogramming, some colonies were partially mechanically plated into individual wells for propagation and further analysis; on day 20, they were stained for alkaline phosphatase (AP) according to a protocol described elsewhere [4].

Immunofluorescent staining was performed as described previously [4]. The following primary antibodies were used for the analysis: SSEA-1 (sc-21702, 1:25), Oct4 (sc-5279, 1:200), and Sox2 (sc-20088, 1:200) (Santa Cruz Biotechnology, USA). Anti-rabbit or anti-mouse immunoglobulin secondary antibodies conjugated with Alexa 488 and Alexa 568 fluorescent dyes (Life Technologies, USA) were used for imaging.

RNA was isolated using a TRIzol reagent (Invitrogen) according to the manufacturer’s protocol. The reverse transcription reaction was performed using 500 ng RNA, reverse transcriptase M-MLV (Invitrogen), and Random Hexamer primers (Thermo Scientific, USA). The prepared cDNA was analyzed on a LightCycler480 device (Roche, Switzerland) using a BioMaster HS-qPCR SYBR Blue kit (Biolabmix, Russia). The amplification reaction was carried out under the following conditions: 95°C for 5 min; 40 cycles of 95°C for 15 s and 60°C for 1 min. Primer sequences are given in *Table 2*.

**Table 2:** Primer sequences for analyzing the expression of pluripotent state markers

Gene	Sequence, 5’–3’
endo-Oct4	CACACTCTACTCGGTCCCTT TGCTTTCAATTCCTCCCCA
endo-Sox2	TATCGAGATAAACATGGCAA CAGAATCAAAACCCAGCAA
endo-Klf4	TCCGATCTACATTTATGACC TTATTGCACATCTGAAACCAC
endo-c-Myc	TCAAAGCCTAACCTCACAA GCAGTTAACATTATGGCTGA
Nanog	TACCTCAGCCTCCAGCAGAT GCAATGGATGCTGGGATACT
Esrrb	GGCGTTCTTCAAGAGAACCA CCCACTTTGAGGCATTTCAT
Tdgf1	TTGGACTTGTTGCTGGGATA CGGAAGGCACAAGCTGGA
Tcl1a	CCGATTAAATATCTCACTCAC TCTCTTATTTCTTGGCATCT
Utf1	TTGCTCCCCAGTCTCTGAAT GAGAAACGGTTTGGTCGAAG
Dnmt3l	AAGACCCATGAAACCTTGAACC GTTGACTTCGTACCTGATGACC
Pecam1	TCCTAAGAGCAAAGAGCAAC TGGGCTTGTCTGTGAATGT
Dppa3	TGGGGAAATCTCTTCTAATTGCT CTTCTAAATCAAACTACCAGGCTT

The search for potential targets was performed using the TargetSpy v1.1 [5], miRanda v3.3a [6], and TargetScan v7.0 [7] software. We selected only the target genes predicted by all three programs and having a reduced expression level in rat ESCs and iPSCs compared to that in fibroblasts. The mRNA expression data were obtained earlier [8].

## RESULTS AND DISCUSSION

**Fig. 1:**
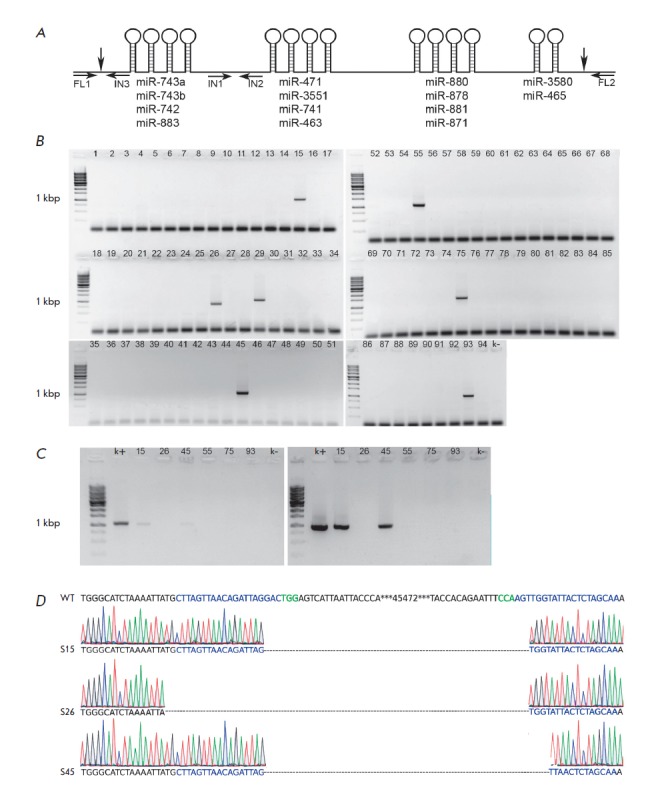
*A *– schematic of the studied miRNA cluster. Horizontal arrows denote PCR primers; vertical arrows denote sites of double strand breaks. *B *– results of the PCR analysis for deletion in subclones. FL1 and FL2 primers were used. *C *– results of the PCR analysis for detection of polyclonal lines (left) and translocation (right) using FL1-IN3 and IN1-IN2 primer pairs, respectively. k+ and k– – positive and negative PCR controls. *D *– examples of Sanger sequencing of PCR products from cells carrying a deletion, using FL1 and FL2 primers. WT – wild type

The studied miRNA cluster is localized in locus 37 of the X chromosome long arm and consists of 14 miRNAs: miR-743a, miR-743b, miR-742, miR-883, miR-471, miR-3551, miR-741, miR-463, miR-880, miR- 878, miR-881, miR-871, miR-3580, and miR-465 (*Fig. 1A*). We tested the hypothesis on the involvement of this miRNA cluster in the reprogramming to a pluripotent state using knockout of these miRNAs, which was induced by deletion of a genome fragment encoding them. A deletion was created using the CRISPR/ Cas9 system with two guide RNAs flanking the locus to be deleted. A total of 94 subcloned lines of male rat fibroblasts were generated; of these, seven lines carried a deletion of the target DNA locus (*Fig. 1B*). The presence of a deletion in the subclones was verified by PCR with flanking primers. In addition, a translocation of the deleted fragment was analyzed using nested primers (*Fig. 1C*). In some lines, the presence of a deletion was confirmed by Sanger sequencing (*Fig. 1D*).

**Fig. 2:**
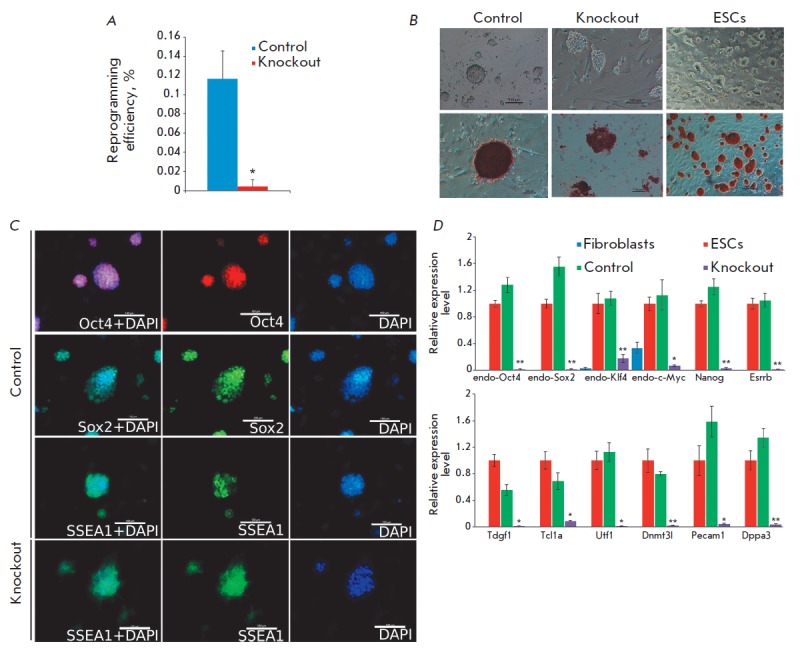
*A *– efficiency of the reprogramming of control and knockout fibroblasts. The star denotes statistically significant differences, *p < 0.05, a Mann-Whitney U-test. *B *– representative images of colonies produced by reprogramming, as well as rat ESCs. Upper panel – phase-contrast, lower panel – staining for AP. *C *– immunofluorescence staining of colonies produced by reprogramming. Scale bar is 100 μm. *D *– RT-PCR analysis of pluripotency state markers. Stars denote statistically significant differences in gene expression between knockout and control cells, *p < 0.05, **p < 0.005, a Student’s t-test

Expression of exogenous pluripotency factors was simultaneously activated in fibroblast lines with a miRNA cluster deletion and in the control cell line. The latter was used for the generation of knockout lines and was electroporated with the pX-458-2sgRNA plasmid not encoding the guide RNAs. The efficiency of the reprogramming of miRNA knockout fibroblasts was significantly lower compared to that of the control line (*Fig. 2A*). During reprogramming, some colonies from both control and experimental wells were partially mechanically transferred for further analysis. The morphology of the cells produced in the control experiment corresponds to that of rat ESCs. These iPSC-like cells are successfully cultured, retain their morphology, and are positively stained for AP after terminating the expression of exogenous pluripotency factors (*Fig. 2B*). They express markers of a pluripotent state, which is confirmed by immunofluorescent staining and real-time RT-PCR (*Fig. 2C,D*).

The cells produced by reprogramming of fibroblasts with knockout of the miR-743a–miR-465 miRNA cluster have an epithelial morphology, which indicates that they have passed the initial reprogramming stage – the mesenchymal-epithelial transition. However, these cells, unlike the control line, form loose colonies. The reprogramming process is incomplete, and the cells die in the absence of doxycycline, which indicates their dependence on the expression of exogenous pluripotency factors. It is worth noting that the cells with knockout of the miR-743a–miR-465 cluster are positively stained for AP and SSEA-1, confirming passage of the initial stages of pluripotency reprogramming (*Fig. 2B,C*). These cells also express pluripotency markers, but their expression level is significantly lower than that in the control group of cells (*Fig. 2D*).

Targets of the studied miRNAs include genes of the TGF-β signaling pathway; its inhibition promotes reprogramming [9]. A significant proportion is represented by genes of the Wnt signaling pathway; its inhibition at early stages is necessary for a successful reprogramming of cells [10]. There are also known reprogramming inhibitors: Cdkn1a and Zeb1 [11, 12].

MiRNAs play an important role in the regulation of various processes, in particular in the reprogramming of cells to a pluripotent state. To date, only a small number of the miRNAs expressed in pluripotent cells and involved in the reprogramming process have been studied. The emergence of genome editing tools has greatly accelerated progress in the study of the functions of both protein-coding genes and non-coding RNAs. Unlike miRNA inhibitors, e.g., on the basis of LNA oligonucleotides, the CRISPR/Cas9 system provides more specific and permanent miRNA knockout. In addition, the use of CRISPR/Cas9 enables knockout of the entire miRNA cluster.

The investigated miRNA cluster is located near the protein-coding gene *Slitrk2*. Similar miRNA clusters have been found in other mammalian species, in particular in mice and humans [13]. These miRNA clusters in different species are supposed to have a common ancestor, but significant differences in the pre-miRNA and seed-region sequences indicate a rapid evolution of these miRNAs [13, 14]. A high expression level of these miRNA clusters was detected in mouse and human testes, and involvement of these miRNAs in the regulation of spermatogenesis in mice was shown bydeletion of some of them [13-15]. The existence of common target genes for these mouse and human miRNAs was functionally confirmed, despite the differences in their nucleotide sequences [14]. It is also worth noting that miRNAs from this cluster are able to functionally compensate for their mutual absence [13]. Unlike mice, the expression level of some miRNAs in rats, in particular miR-741, is comparable in testes and pluripotent cells, which may indicate the species-specific features of pluripotent rat cells [3, 15]. However, a huge pool of potential target genes may comprise to common genes involved in the reprogramming process in different species. Therefore, this miRNA cluster may be involved in the reprogramming of not only rat cells, but this issue requires further study.

Disruption of the reprogramming process upon deletion of a DNA fragment containing a cluster of 14 miRNA (miR-743a through miR-465) suggests that all or some of them are involved in this process. It is worth noting that deletion of this large-sized fragment might affect either unknown regulatory elements or non-annotated genes. In any case, our study may be considered as a first step in the investigation of this miRNA cluster during the reprogramming of cells to a pluripotent state.

## Abbreviations

iPSC: induced pluripotent stem cell
miRNA: microRNA
ESC: embryonic stem cell
CRISPR: clustered regularly interspaced short palindromic repeat
PAM: protospacer adjacent motif
RT-PCR: reverse transcription polymerase chain reaction
AP: alkaline phosphatase
